# Effect of Dexamethasone on Abiraterone Pharmacokinetics in Mice: Determined by LC/MS Analysis

**DOI:** 10.3390/medicines10030021

**Published:** 2023-03-06

**Authors:** Subrata Deb, Mohamed Ben-Eltriki, Hans Adomat, Mei Y. Chin, Emma S. Tomlinson Guns

**Affiliations:** 1 Department of Pharmaceutical Sciences, College of Pharmacy, Larkin University, Miami, FL 33169, USA; 2 The Vancouver Prostate Centre at Vancouver General Hospital, 2660 Oak Street, Vancouver, BC V6H 3Z6, Canada; 3 Department of Pharmacology and Therapeutics, Max Rady College of Medicine, University of Manitoba, Winnipeg, MB R3E 0T6, Canada

**Keywords:** abiraterone, interaction, dexamethasone, cytochrome P450, liquid chromatography–mass spectrometry

## Abstract

**Background:** Abiraterone acetate is a cytochrome P450 17A1 (CYP17A1) inhibitor that is indicated for use in both castration-resistant and castration-sensitive prostate cancer patients. To manage the mineralocorticoid effects of CYP17A1 inhibition, a glucocorticoid such as dexamethasone is co-administered with abiraterone. The goal of the present study was to understand the effect of dexamethasone on the disposition of abiraterone. **Methods:** Adult male CD-1 mice were treated with either dexamethasone (80 mg/kg/day) or vehicle for three consecutive days, followed by the administration of a single dose of abiraterone acetate (180 mg/kg) as an oral gavage. Blood samples were collected by tail bleeding at timepoints between 0 to 24 h. Subsequently, abiraterone was extracted from the mouse serum using a neutral pH condition and serum abiraterone levels were determined using a liquid chromatography–mass spectrometry assay. **Results:** Our results demonstrated that dexamethasone lowered the maximum plasma concentration and area under the curve parameters by approximately five- and ten-fold, respectively. Similar effects were also observed on the plasma half-life and oral clearance parameters. This is the first report of dexamethasone effect on abiraterone disposition in vivo. **Conclusions:** We conclude that dexamethasone has the potential to reduce the plasma abiraterone level and thus compromise its CYP17A1 inhibitory ability in the procancerous androgen biosynthesis pathway. Thus, use of a higher abiraterone dose may be warranted when used alongside dexamethasone.

## 1. Introduction

Prostate cancer (PCa) is the most commonly diagnosed cancer in men after lung cancer, and androgens are the major driving force of PCa [1]. Abiraterone is an orally administered drug indicated for both metastatic castration-resistant prostate cancer (CRPC) and metastatic high-risk castration-sensitive prostate cancer (CSPC) [2]. The therapeutic action of abiraterone stems from the inhibition of cytochrome P450 17α-hydroxylase/C17, 20-lyase (CYP17A1), and, eventually, blocking the formation of the most potent androgen receptor ligands are such testosterone and dihydrotestosterone [3,4]. Since CYP17A1 is a key enzyme in the gonadal and extragonadal steroid biosynthesis, abiraterone has a wide spectrum of adverse effects including hypertension, edema, and hypokalemia [2]. The diversion of steroid precursors, as a result of CYP17A1 inhibition, to the mineralocorticoid pathway is responsible for some of these adverse effects [5]. Interestingly, abiraterone also works as a partial antagonist of the androgen receptor, which is the most powerful driver of PCa [6]. Although therapeutically very successful and considered, a few years after the introduction of this anticancer drug to the clinic, resistance to abiraterone has been observed [7]. A multitude of mechanisms, including altered androgen receptor activation and by-pass mechanisms of steroid formation, have been proposed to explain this resistance phenomenon [6,7].

Abiraterone is administered as a prodrug in the acetate ester form and is hydrolyzed to the active drug through yet to be discovered esterase enzymes [8]. The non-acetylated form of abiraterone undergoes CYP3A4- and sulfotransferase 2A1 (SULT2A1)-mediated phase I and phase II metabolism, respectively. The drug is highly protein bound (>99%), and about half of the administered acetate drug is excreted unchanged through feces [2,8,9]. In vivo studies indicate that CYP2D6 substrates with narrow therapeutic index are susceptible to abiraterone-mediated inhibition [10]. Similarly, CYP3A4 and CYP2C8 activities are potentially inhibited by abiraterone [9,11,12], however, there is very limited in vivo evidence available to corroborate these inhibitory actions. Interestingly, abiraterone can inhibit the CYP3A4-mediated hepatic and intestinal metabolism of calcitriol in vitro [11]. Similarly, abiraterone can also inhibit P-glycoprotein and OATP1B1, but it is not a substrate of the efflux transporters [2].

Dexamethasone is a commonly used glucocorticoid for numerous health conditions including inflammation, nausea, and vomiting [13,14]. Because of its versatile mechanism of action, dexamethasone is commonly used in the cancer treatment regimens as co-medication and premedication to manage some of the treatment outcomes (e.g., pain, nausea, or vomiting) or comorbidities [13]. The use of dexamethasone with abiraterone has been advocated in recent times due to its ability to address some of the challenges related to abiraterone resistance and to mitigate the CYP17A1-associated mineralocorticoid adverse effects [6,15,16]. The antiangiogenic effects of dexamethasone through inhibition of interleukin-6 and vascular endothelial growth factor can provide advantages in CRPC treatment [17]. In relation to biotransformation, dexamethasone is an agonist of the pregnane X receptor (PXR) and glucocorticoid receptor [18], and thus has the ability to induce CYP3A and CYP2C isoforms in rodent and human drug metabolism models. In addition, phase II enzymes have also been reported to be induced by dexamethasone [19]. Though the use of dexamethasone with abiraterone is encouraged, there is no knowledge of the effect of dexamethasone on abiraterone disposition available. The purpose of the present study was to determine if dexamethasone has any effect on the oral bioavailability of abiraterone in mice.

## 2. Materials and Methods

### 2.1. Test Compounds and Materials

Abiraterone acetate powder was generously gifted by Johnson & Johnson (New Brunswick, NJ, USA). Dexamethasone sodium was obtained from Sigma-Aldrich Canada Ltd. (Oakville, ON, Canada). Dexamethasone solutions were prepared in a water/ethyl alcohol solution, whereas abiraterone oral gavage was prepared in benzyl alcohol. All other chemicals were obtained from commercial sources.

### 2.2. Single Dose Pharmacokinetic Study Design

**Figure 1:**
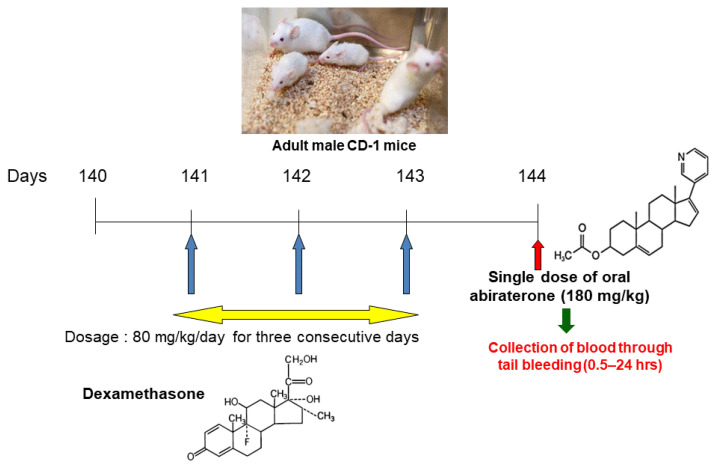
Study design involving treatment of adult male CD-1 mice with intraperitoneal dexamethasone followed by a single oral dose of abiraterone acetate.

Adult male CD-1 mice were obtained from Harlan Laboratories Inc. (Montreal, QC, Canada). Mice were housed in polycarbonate cages with corn-cob bedding at a temperature of 20–23 °C and had a 12 h photoperiod. Mice were given a commercial mouse diet and water ad libitum. Mice were cared for and treated in accordance with the University of British Columbia Committee on Animal Care. Six mice were treated with dexamethasone at a dosage of 80 mg/kg body weight or an equivalent volume of vehicle (three mice per group) by intraperitoneal injection once a day, for three consecutive days. On the fourth day, each mouse received a single dose of abiraterone acetate oral gavage at a dosage of 180 mg/kg (Figure 1). A total of 50 µL of blood samples was collected by tail bleeding at 0, 0.5, 1, 2, 4, 6, 8, 12, and 24 h following abiraterone acetate administration. Mice were euthanized 24 h after the administration of abiraterone acetate. The blood samples were centrifuged at 3000 rpm for 10 min and the supernatant serum was placed into Eppendorf tubes, which were stored at −20 °C pending analysis by liquid chromatography–mass spectrometry (LC/MS).

### 2.3. Serum Extraction and Sample Preparation

Deuterated testosterone (d3T, 10 ug/mL) was used as an internal standard (IS) in the assay. A total of 2 µL IS stock (d3T) was added to 8 µL extracted serum and, subsequently, the acetonitrile precipitation method was used to extract abiraterone and d3T from the serum. The samples were vortex mixed for 5 min and centrifuged at 20,000 rpm for 5 min at 4 °C. The supernatant was transferred to the inserts for injection into the LC/MS. pH variation experiments were carried out to optimize the extraction of abiraterone at acidic (1M HCl), basic (1M NaOH), or neutral pH ranges.

### 2.4. Quantification of Abiraterone by LC/MS Method

A Waters Acquity Ultraperformance liquid chromatography (UPLC) system coupled to a Quattro Premier XE triple quadrupole mass spectrometer (MS) controlled by MASSLYNX version 4.1 software (Waters, Milford, MA, USA) was used for quantification of the abiraterone, abiraterone acetate, and internal standard (d3T). Chromatographic separations of the analytes were carried out on a Waters Acquity^TM^ UPLC BEH C_18_ column (2.1 × 100 mm, 1.7 µM) at 40 °C with a flow rate maintained at 0.3 mL/min and a total run time of 7 min. The acetonitrile gradient (with 0.1% formic acid) mobile had with the following conditions: 40% (0–0.2 min), followed by 40–98% (0.2–3 min), 98% (3–5 min), and, finally, 40% (5.1–7 min). The LC eluant was introduced into the MS and all data were collected in electrospray ionization positive (ESI+) mode with a capillary voltage of 3.2 kV. Source and desolvation temperatures were 120 °C and 300 °C, respectively, and N_2_ gas flow was 1000 L/h. Data acquisition was performed by multiple reaction monitoring using *m*/*z* 350.3 > 156.2 and *m*/*z* 350.3 > 334.3 transitions for abiraterone (cone/collision voltages 90/52 and 90/37, respectively), an *m*/*z* 392.3 > 332.3 transition for the abiraterone acetate (cone/collision voltages 50/35), and an *m*/*z* 292.3 > 97 transition for d3T (cone/collision voltages 32/21). Quantitative data processing was with QuanLynx (Waters) using an area under the peak or curve (AUC) of the analyte/internal standard versus a calibration series and exported to Excel for further analysis.

### 2.5. Data Analyses

Microsoft Excel (version Microsoft 365; Microsoft Corp., Redmond, WA, USA) was used to enter the data and analyze them. The comparison between the pharmacokinetic parameters was carried out using non-compartmental analyses. The maximum serum concentration (Cmax) and time taken to reach maximum concentration (Tmax) were calculated straight from the tabulated numbers. The trapezoidal method was used to compute the AUC_0–24 h_ parameter. Dose normalization of AUC_0–24 h_ was achieved by dividing the raw AUC parameter with the dose administered per kilogram body weight. A Student *t*-test (parametric) or the Mann–Whitney test (nonparametric unpaired *t*-test) was used to compare between two treatment groups. A two-tailed *p* value with the level of significance was set a priori at *p* < 0.05.

## 3. Results

### 3.1. LC/MS Analysis

**Figure 2:**
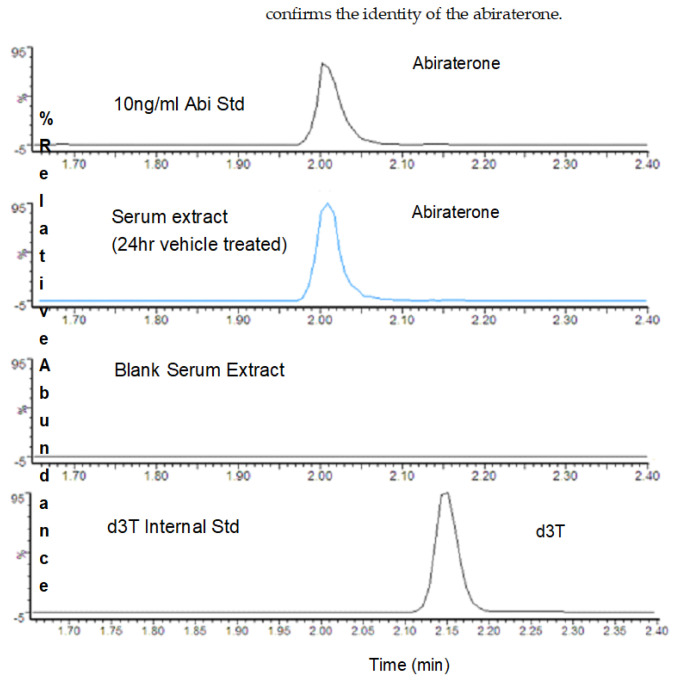
Representative LC/MS chromatogram showing abiraterone and deuterated testosterone internal standard. Chromatograms were obtained either from the standard solutions were from serum extracted samples as indicated in the labels.

The extractions of abiraterone and internal standard from the mice serum were maximum at a neutral pH without intervention of pH modulators. An LC/MS-based assay was developed to estimate the abiraterone and internal standard levels. In the present experimental conditions, the abiraterone peak (*m*/*z* 350.3 > 156.2 and *m*/*z* 350.3 > 334.3) either from the standard solution or spiked serum eluted at 2.00 min (Figure 2). The d3T internal standard (*m*/*z* 292.3 > 97) eluted around a similar time of 2.15 min. Very little abiraterone acetate (*m*/*z* 392.3 > 332.3), the parent compound administered to mice, was detected in the LC/MS analyses. The overlay of abiraterone standard peaked with the serum-extracted peak.

### 3.2. Effect of Dexamethasone on Abiraterone Pharmacokinetics

**Figure 3:**
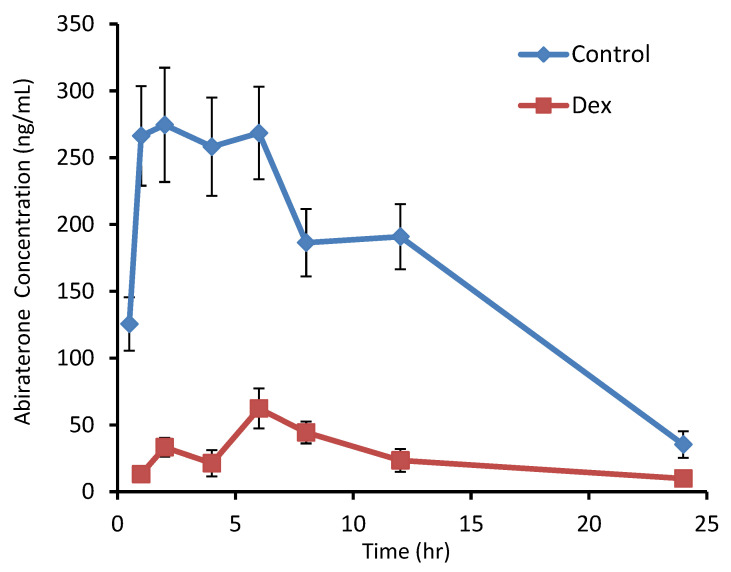
Serum concentration-time curves of abiraterone acetate (180 mg/kg) following a single oral administration to adult male CD-1 mice (*n* = 3) in the presence or absence (vehicle) of intraperitoneal dexamethasone (80 mg/kg/day). Data are expressed as mean ± SEM (n of 3 for each time point).

Abiraterone has relatively lower bioavailability with a maximum plasma concentration of 271.4 ng/mL and area under the curve (AUC_0–24_) of 3769.9 ng.h/mL. Interestingly, it reaches maximum plasma concentration at a relatively shorter time of 1.9 h. Pre-treatment of mice with dexamethasone for three days altered the abiraterone pharmacokinetics (Figure 3). Cmax levels were five times lower following dexamethasone treatment compared to vehicle-treated mice (Table 1). Comparatively, there was approximately a 10-fold difference in abiraterone AUC between the two groups. Similar effects of dexamethasone treatment were also observed on the plasma half-life, which was significantly decreased following pretreatment. Our analysis indicates an approximate 10-fold increase in apparent oral clearance (CL/F) in mice treated with dexamethasone compared to vehicle-treated mice.

## 4. Discussion

Abiraterone is a CYP17A1 inhibitor approved for both CSPC and CRPC [2,6]. In spite of its pivotal role in the treatment of PCa, the interactions related to abiraterone are not well studied. Dexamethasone is a commonly used glucocorticoid used for numerous conditions including cancer [13]. In recent times, the use of dexamethasone with abiraterone has been advocated [6,15,16]. The goal of the present study was to understand the interaction potential between dexamethasone and abiraterone.

A single dose of abiraterone acetate was administered to adult mice after treating them with dexamethasone for three consecutive days. The extraction of abiraterone from serum samples in neutral pH conditions was the most effective way of preparing the samples and eventually quantitative determination using an LC/MS assay. From very little or no detection of the parent abiraterone acetate, it can be inferred that mouse esterase enzymes are able to fully catalyze the bioactivation of the prodrug. Consistent with humans, the abiraterone is poorly absorbed in mice. Dexamethasone pretreatment led to a significant decrease in abiraterone Cmax and AUC. It also expedited abiraterone excretion from the central compartment as indicated by decreased plasma half-life and increased oral clearance.

Abiraterone acetate is a prodrug, which is converted to abiraterone by esterase enzymes. CYP3A4 and SULT2A1 are the key enzymes responsible for the formation of N-oxide abiraterone sulfate and its intermediate(s) [2,8,9]. Due to the age group of people inflicted with CRPC, several medications are often used in these patients to manage their comorbidities [20]. Both CYP3A4 and SULT2A1 can be modulated by xenobiotics and thus have the potential to alter the disposition of their substrates. Dexamethasone is a CYP3A4 and SULT2A1 inducer [18,19] and has the potential to alter the systemic exposure to abiraterone. Bernard et al. (2015) reported that coadministration of rifampin, a CYP inducer, with abiraterone, led to 45% decrease in plasma abiraterone levels. However, ketoconazole, a known CYP3A4 inhibitor, did not alter disposition of abiraterone in humans [9]. This raises the potential that increased SUL2A1 may be potentially contributing to the lower plasma concentration of abiraterone following dexamethasone treatment, along with altered CYP3A4. It is well known that there are numerous CYP3A modulators [11,19,21], whereas the number of SULT inducers and inhibitors appears to be limited. Thus, CYP3A may be more frequently affected and contribute to the altered disposition of abiraterone. However, there are very limited experimental abiraterone DDI reports available at this time. A review of the electronic charts of patients undergoing abiraterone treatment indicated that a wide variety of medications, including cardiovascular, opioid analgesics, anticoagulants, and antiresorptive, is ingested by the PCa patients [20]. It is consequential to note that abiraterone has a poor bioavailability and is also strongly affected by food intake [22]. As with all poorly absorbed drugs, even a minor change in the plasma profile could have a significant effect on the therapeutic outcome of the victim drug. The use of glucocorticoids such as dexamethasone is indicated to manage mineralocorticoids-related adverse effects [15,16,23]. While it is advantageous to administer dexamethasone with abiraterone also for its intrinsic anti-inflammatory and antiangiogenic effect [17], the biotransformation related effects of dexamethasone should not be ignored. Especially because it is a ligand of two nuclear receptors known to induce multiple phase I and phase II enzymes [18].

## 5. Conclusions

In our knowledge, this is the first report showing the effect of dexamethasone on abiraterone disposition in vivo. We have described that plasma concentration and half-life of abiraterone are significantly lowered by this commonly used glucocorticoid. This could have a significance on the anticancer efficacy of abiraterone as lower abiraterone concentration can lead to diminished CYP17A1 inhibition and higher procancerous androgen formation as a potential consequence. A higher abiraterone concentration may be warranted when used along with dexamethasone. Future studies need to focus on the pharmacodynamic implications of the altered abiraterone disposition in PCa treatment and abiraterone dose adjustment required to counteract the inducing effects of dexamethasone.

## Figures and Tables

**Table 1:** Pharmacokinetic parameters of abiraterone following a single oral dose of abiraterone acetate (180 mg/kg) administered to adult male CD-1 mice (*n* = 3) in the presence or absence (vehicle) of intraperitoneal dexamethasone (80 mg/kg/day).

PK Parameters	Control	Dexamethasone
Cmax (ng/mL)	271.4	53.3 *
Tmax (h)	1.9	4.2 *
AUC_0–24 h_ (ng.h/mL)	3769.9	383.1 *
T_1/2_ (h)	13.7	10.2 *
Oral CL (h/L)	2.2	21.7 *

* Significantly different than the control group, *p* < 0.05.

## Data Availability

The data presented in this study are available on reasonable request from the corresponding authors.

## References

[1] Worldwide Cancer Research Fund International Worldwide Cancer Data. https://www.wcrf.org/cancer-trends/worldwide-cancer-data/.

[2] Janssen Biotech Inc ZYTIGA® (Abiraterone Acetate). https://www.zytiga.com/.

[3] Beckett R.D., Rodeffer K.M., Snodgrass R. (2012). Abiraterone for the treatment of metastatic castrate-resistant prostate cancer. Ann. Pharmacother..

[4] Fizazi K., Scher H.I., Molina A., Logothetis C.J., Chi K.N., Jones R.J., Staffurth J.N., North S., Vogelzang N.J., Saad F. (2012). Abiraterone acetate for treatment of metastatic castration-resistant prostate cancer: Final overall survival analysis of the COU-AA-301 randomised, double-blind, placebo-controlled phase 3 study. Lancet Oncol..

[5] E Ang J., Olmos D., De Bono J.S. (2009). CYP17 blockade by abiraterone: Further evidence for frequent continued hormone-dependence in castration-resistant prostate cancer. Br. J. Cancer.

[6] Thakur A., Roy A., Ghosh A., Chhabra M., Banerjee S. (2018). Abiraterone acetate in the treatment of prostate cancer. Biomed. Pharmacother..

[7] Mostaghel E.A., Marck B.T., Plymate S.R., Vessella R.L., Balk S., Matsumoto A.M., Nelson P.S., Montgomery R.B. (2011). Resistance to CYP17A1 inhibition with abiraterone in castration-resistant prostate cancer: Induction of steroidogenesis and androgen receptor splice variants. Clin. Cancer Res..

[8] Schultz H.B., Meola T.R., Thomas N., Prestidge C.A. (2020). Oral formulation strategies to improve the bioavailability and mitigate the food effect of abiraterone acetate. Int. J. Pharm..

[9] Bernard A., Vaccaro N., Acharya M., Jiao J., Monbaliu J., De Vries R., Stieltjes H., Yu M., Tran N., Chien C. (2015). Impact on abiraterone pharmacokinetics and safety: Open-label drug-drug interaction studies with ketoconazole and rifampicin. Clin. Pharmacol. Drug Dev..

[10] Chi K.N., Tolcher A., Lee P., Rosen P.J., Kollmannsberger C.K., Papadopoulos K.P., Patnaik A., Molina A., Jiao J., Pankras C. (2013). Effect of abiraterone acetate plus prednisone on the pharmacokinetics of dextromethorphan and theophylline in patients with metastatic castration-resistant prostate cancer. Cancer Chemother. Pharmacol..

[11] Deb S., Chin M.Y., Adomat H., Guns E.S.T. (2014). Abiraterone inhibits 1alpha,25-dihydroxyvitamin D3 metabolism by CYP3A4 in human liver and intestine in vitro. J. Steroid Biochem. Mol. Biol..

[12] Monbaliu J., Gonzalez M., Bernard A., Jiao J., Sensenhauser C., Snoeys J., Stieltjes H., Wynant I., Smit J.W., Chien C. (2016). In Vitro and In Vivo Drug-Drug Interaction Studies to Assess the Effect of Abiraterone Acetate, Abiraterone, and Metabolites of Abiraterone on CYP2C8 Activity. Drug Metab. Dispos..

[13] Cook A.M., McDonnell A.M., Lake R.A., Nowak A.K. (2016). Dexamethasone co-medication in cancer patients undergoing chemotherapy causes substantial immunomodulatory effects with implications for chemo-immunotherapy strategies. Oncoimmunology.

[14] Rhen T., Cidlowski J.A. (2005). Antiinflammatory action of glucocorticoids--new mechanisms for old drugs. N. Engl. J. Med..

[15] Roviello G., Sobhani N., Corona S.P., D’Angelo A. (2020). Corticosteroid switch after progression on abiraterone acetate plus prednisone. Int. J. Clin. Oncol..

[16] Van Praet C., Fonteyne V., Lumen N. (2022). Dexamethasone use in metastatic castration-resistant prostate cancer patients treated with abiraterone acetate: This “cort” is not out of order!. Asian J. Androl..

[17] Kassi E., Moutsatsou P. (2011). Glucocorticoid receptor signaling and prostate cancer. Cancer Lett..

[18] Rushmore T.H., Kong A.N. (2002). Pharmacogenomics, regulation and signaling pathways of phase I and II drug metabolizing enzymes. Curr. Drug Metab..

[19] Xu C., Li C.Y.-T., Kong A.-N.T. (2005). Induction of phase I, II and III drug metabolism/transport by xenobiotics. Arch. Pharm. Res..

[20] Jamani R., Lee E.K., Berry S.R., Saluja R., DeAngelis C., Giotis A., Emmenegger U. (2016). High prevalence of potential drug-drug interactions in patients with castration-resistant prostate cancer treated with abiraterone acetate. Eur. J. Clin. Pharmacol..

[21] Deb S., Pandey M., Adomat H., Guns E.S.T. (2012). Cytochrome P450 3A-mediated microsomal biotransformation of 1alpha,25-dihydroxyvitamin D3 in mouse and human liver: Drug-related induction and inhibition of catabolism. Drug Metab. Dispos..

[22] Solymosi T., Ötvös Z., Angi R., Ordasi B., Jordán T., Molnár L., McDermott J., Zann V., Church A., Mair S. (2017). Novel formulation of abiraterone acetate might allow significant dose reduction and eliminates substantial positive food effect. Cancer Chemother. Pharmacol..

[23] Dizdar O. (2015). Is dexamethasone a better partner for abiraterone than prednisolone?. Oncologist.

